# Fifth District Medical Society

**Published:** 1904-03

**Authors:** 


### Fifth District Medical Society.

Representatives from sixteen counties of this district met at San Antonio, February 2d. Dr. Malone Duggan, Eagle Pass, Maverick county, was elected temporary chairman, and Dr. Marvin B. Grace, Seguin, Guadalupe county, temporary secretary. After Dr. Witten B. Russ, San Antonio, Councilor for the District, had stated the object of the meeting, a committee, consisting of Drs. Alonzo Garwood, New Braunfels, Comal county, and John V. Spring and Witten B. Russ, of San Antonio, Bexar county, was appointed to draft a constitution and by-laws. At the evening session this committee made its report, and presented a constitution and by-laws, which were adopted. The following officers were elected: President, Dr. Malone Duggan, Eagle Pass, Maverick county; Vice-Presidents, Drs. J. R. Evans, Devine, Medina county, William S. Pickett, Karnes City, Karnes county, Charles W. Watt Uvalde, Uvalde county, Henry Leonard, New Braunfels, Comal county, David H. Houston, Floresville, Wilson county, Asa M. Stamps, Seguin, Guadalupe county, and R. N. Lane, Eagle Pass, Maverick county; Secretary, Dr. Marvin B. Grace, Seguin, Guadalupe county; Treasurer, Dr. John T. Fitzsimon, Castroville, Medina county, and Censors, Drs. Alonzo Garwood, New Braunfels, Comal county, W. A. King, Falls City, Karnes county, and William Myers, Seguin, Guadalupe county.

